# Prevalência da Cardiomiopatia Hipertrófica no Brasil: Qual é a nossa Realidade?

**DOI:** 10.36660/abc.20260100

**Published:** 2026-03-18

**Authors:** Silvia Moreira Ayub-Ferreira

**Affiliations:** 1 Universidade de São Paulo São Paulo SP Brasil Universidade de São Paulo, São Paulo, SP – Brasil; 2 Hospital Sírio-Libanês São Paulo SP Brasil Hospital Sírio-Libanês, São Paulo, SP – Brasil

**Keywords:** Cardiomiopatia Hipertrófica, Ecocardiograma, Epidemiologia

A cardiomiopatia hipertrófica (CMH) é uma doença cardíaca hereditária cuja prevalência é subestimada em diversas regiões do mundo. No Brasil, esse desafio também é significativo, especialmente diante da heterogeneidade da população. Nesta edição, destacamos um estudo pioneiro que investigou a ocorrência do fenótipo ecocardiográfico da CMH na coorte ELSA-Brasil.^1^

Para quem não conhece, o Estudo Longitudinal Brasileiro de Saúde do Adulto (ELSA-Brasil) é um amplo estudo de coorte multicêntrico, concebido para investigar os fatores de risco para obesidade, diabetes e doenças cardiovasculares em servidores públicos brasileiros de grandes centros urbanos, com idades entre 35 e 74 anos.^2^

A subanálise apresentada nesta publicação incluiu mais de três mil indivíduos, a maioria com 60 anos ou mais, submetidos a ecocardiografia transtorácica. Um fenótipo definitivo da CMH, definido como espessamento assimétrico da parede do ventrículo esquerdo superior a 15 mm ou hipertrofia apical inequívoca com espessura da parede superior à dos segmentos basais^3^ foi identificado em aproximadamente 1,5 indivíduos por mil.

Vale ressaltar que essa prevalência pode estar subestimada, uma vez que a amostra incluiu apenas adultos mais velhos, enquanto a faixa etária mais frequente de apresentação da doença é entre adultos jovens e de meia-idade, na faixa dos 20 aos 40 anos.^4,5^

Em investigações epidemiológicas anteriores, baseadas em triagem ecocardiográfica, a prevalência de CMH na população geral foi estimada em aproximadamente 1 em 500 indivíduos. No entanto, estudos mais recentes, incluindo indivíduos portadores de variantes patogênicas — particularmente indivíduos com genótipo positivo e fenótipo negativo — sugerem uma prevalência maior, próxima a 1 em 200 indivíduos.^4^

Outro ponto importante foi a prevalência de condições como hipertensão, obesidade e distúrbios metabólicos. Esses achados levantam questões sobre a influência dos ambientes metabólico e hemodinâmico na expressão da hipertrofia miocárdica.^6^

Com o surgimento de terapias específicas para a CMH, se torna ainda mais crucial compreender quem são esses pacientes e como eles se apresentam no Brasil.^4^ Assim, este artigo contribui para a compreensão da CMH em nosso contexto e reforça o papel fundamental de iniciativas de longo prazo como o ELSA-Brasil,^2^ para que possamos traduzir a ciência em políticas de saúde, diagnósticos oportunos e melhoria da qualidade de vida.
