# Supplementary material for: Prevalência da Cardiomiopatia Hipertrófica no Brasil: Qual é a nossa Realidade?
Source: Arq Bras Cardiol. 2026 Mar 18;123(1):e20260100. [Article in Portuguese] doi: 10.36660/abc.20260100 (PMC13127811; doi:10.36660/abc.20260100)
Supplement: Translation

# Translation: Prevalence of Hypertrophic Cardiomyopathy in Brazil: What is Our Reality?

**DOI:** 10.36660/abc.20260100i

**Authors:** Silvia Moreira Ayub-Ferreira

**Affiliations:** 1 São Paulo SP Brazil Universidade de São Paulo, São Paulo, SP – Brazil; 2 São Paulo SP Brazil Hospital Sírio-Libanês, São Paulo, SP – Brazil

**Keywords:** Hypertrophic Cardiomyopathy, Echocardiogram, Epidemiology

Hypertrophic cardiomyopathy (HCM) is a hereditary heart disease whose prevalence is underestimated in several regions of the world. In Brazil, this challenge is also significant, especially given the country's population heterogeneity. In this issue, we highlight a pioneering study that investigated the occurrence of the echocardiographic phenotype of HCM in the ELSA-Brasil cohort.1

For those unfamiliar, the Brazilian Longitudinal Study of Adult Health (ELSA-Brasil) is a large, multicenter cohort study designed to investigate risk factors for obesity, diabetes, and cardiovascular diseases in Brazilian public servants from large urban centers, aged 35 to 74 years.^2^

The sub-analysis presented in this publication included more than three thousand individuals, most of whom were aged 60 years or older and underwent transthoracic echocardiography. A definitive HCM phenotype, defined as asymmetric left ventricular wall thickness greater than 15 mm, or unequivocal apical hypertrophy with wall thickness exceeding that of the basal segments^3^ was identified in approximately 1.5 individuals per thousand.

It is worth noting that this prevalence may be underestimated, since the sample included only older adults, whereas the most frequent age of presentation is among young and middle-aged adults in their 20s to 40s.^4,5^

In earlier epidemiological investigations based on echocardiographic screening, the prevalence of HCM in the general population was estimated at approximately 1 in 500 individuals. However, more recent studies incorporating individuals carrying pathogenic variants—particularly genotype-positive, phenotype-negative subjects—have suggested a higher prevalence, approaching 1 in 200 individuals.^4^

Another important point was the prevalent conditions such as hypertension, obesity, and metabolic disorders. These findings raise questions about the influence of metabolic and hemodynamic environments on the expression of myocardial hypertrophy.^6^

With the emergence of specific therapies for HCM, understanding who these patients are and how they present in Brazil becomes even more crucial.^4^ Thus, this article contributes to the understanding of HCM in our context and reinforces the fundamental role of long-term initiatives such as ELSA-Brasil,^2^ so that we can translate science into health policies, timely diagnosis, and improved quality of life.
